# Flexible emotional regulation typology: associations with PTSD symptomology and trait resilience

**DOI:** 10.1186/s40359-024-01573-4

**Published:** 2024-02-16

**Authors:** Eric Spikol, Emily McGlinchey, Martin Robinson, Cherie Armour

**Affiliations:** 1 Stress Trauma and Related Conditions (STARC) Research Centre, School of Psychology, Queen’s University Belfast, David Keir Building, 18-30 Malone Road, BT9 5BN Belfast, Northern Ireland, UK; 2 School of Psychology, Queen’s University Belfast, David Keir Building, 18-30 Malone Road, BT9 5BN Belfast, Northern Ireland, UK

**Keywords:** Resilience, PTSD, Emotion regulation, Flexibility, Emotion regulation sequence

## Abstract

**Background:**

Multiple factors influence posttraumatic stress disorder (PTSD) risk in trauma exposed individuals. An established association exists between trait resilience and decreased PTSD distress and between emotion regulation (ER) ability/flexibility and trait resilience. Typologies in ER ability/flexibility, associated with trait resilience and PTSD experience, could explain the difference in risk. This study aimed to explore the relationship between ER ability, ER flexibility, context sensitivity, resilience, and PTSD.

**Methods:**

Data from *N* = 563 trauma exposed UK residents was used in a latent profile analysis (LPA) and membership in the resultant profiles was explored in a logistic regression of sociodemographics, resilience, and PTSD symptomology.

**Results:**

Analysis showed 2 latent profiles (High Flexibility, Low Flexibility) typified by emotion regulation ability and context sensitivity. Members of the Low Flexibility profile were more likely to be younger, male, endorsing less trait resilience, and experiencing negative cognition/mood and hyperarousal PTSD symptomology.

**Conclusions:**

Difficulties in ER ability and flexibility could be improved with targeted learning in a therapeutic or home setting, potentially increasing trait resilience after trauma exposure and reducing PTSD distress.

**Supplementary Information:**

The online version contains supplementary material available at 10.1186/s40359-024-01573-4.

## Introduction

Exposure to trauma is the primary criteria of posttraumatic stress disorder (PTSD), considered necessary for a clinical diagnosis and seen as a catalyst for distress. However, not all trauma exposed individuals become traumatised [1–2] and not all traumatised individuals have the same experience of PTSD symptomology [3–4]. While there is an established association between higher resilience and decreased experiences of PTSD symptomology [5–7], there remains a popular conception of ‘resilience’ as an absolute typology; you have it, or you don’t. Recent research has challenged this idea, describing post-trauma resilience as the use of multiple strategies from an individual ‘portfolio’ [8–10], a multi-system model of interconnecting factors [11], and the result of an adaptive process involving situational assessment, reaction, and reaction strategy management [12–15]. These novel paradigms are better descriptors of resilience as a fluid mechanism rather than a static trait which would need to function in the same manner regardless of the situation.

Clearly, variation in resilience exists, but simply rephrasing the language around resilience from a binary typology to a high/moderate/low spectrum of typologies would not solve the issue. Resilience has been hard to define as its complexity makes it difficult to absolutely quantify and measure as it cannot be directly observed, only inferred from a range of associated mechanisms and individual outcomes [16–18]. Resilience typologies along a high/moderate/low spectrum would imply a static construct functioning in the same way in all situations, with the only difference between individuals being their level of ability; however, given the range of factors influencing the individual before, during, and after trauma, this seems unlikely. It may be useful, therefore, to explore typologies in contributory factors by examining their impact on resilience and PTSD experience concurrently.

One important factor in exploring the impact of ‘resilience’ on PTSD distress is the contribution of emotion regulation, the ability of an individual to adapt their experience and conveyance of emotion depending on the situation, environment, or other factors [12]. The sequential model of emotion regulation (ER) as a facilitator of resilience, focuses on flexibility and situational adaptation both during and after the traumatic event [7]. This is important, as the circumstances during and after these events are likely to be chaotic and mercurial, requiring flexibility and adaptation on a potential moment-by-moment basis for effective resilience [15, 19]. In this model, an individual assesses the contextual cues of the situation (context sensitivity), selects and utilises an appropriate coping strategy (repertoire), and adjusts/changes their strategy based on its effectiveness in the situation (feedback monitoring). Missing the presence or absence of contextual cues can lead to a maladaptive or insufficient response, applying an ineffective coping strategy may result in the inability to cope with the stressor, and inflexibility in feedback monitoring can leave an individual rigidly adhering to an inappropriate strategy; all of which increase distress and the likelihood of PTSD symptomatology [20–22]. Robinson and colleagues [15] have described this sequence as beginning with the initial traumatic event and the presence/absence of context from this environment, for example, sources of threat/urgency or the roles of others present during the event. These inform an individual’s immediate emotional reaction. Following the event and based on contextualisation, individuals will rely on a coping strategy and having more strategies ‘on hand’ to choose from can assist them in selecting one appropriate to the situation. Finally, an individual needs to be able to recognise when then have selected an unsuccessful, maladaptive, or inappropriate coping strategy (alcohol/drugs misuse, etc.) and adjust to a new, hopefully successful coping strategy.

Indeed, there is an established association between ER flexibility and higher levels of post-trauma resilience [12, 23–24], not only demonstrating the association between ER flexibility and reduced post-trauma distress/reduced PTSD risk, but also suggesting that as ER flexibility can affect ‘resilience’ as it has been measured. It must be noted, however, that this fluid process has been conceptualised, measured, and operationalised in several ways and using several techniques [7, 12–15, 19−22]. This study utilised a cross-sectional survey-based design to test the relationship between ER flexibility and resilience typology based on previous ER flexibility sequence research and simplicity of replication in larger or specific populations.

PTSD pathology is typically screened using either total symptom burden, or endorsement of the distinct symptoms comprising this disorder [25]. The items can be broken down into four symptom cluster subscales in keeping with the DSM-5 conceptualisation of PTSD: re-experiencing/intrusion, avoidance, negative cognition/mood, and hyperarousal. Differences in symptom experience have been shown to vary in association with a variety of factors including brain physiology [26–27], substance misuse [28–29], and gender [30–31]. These differences have been found in trauma-exposed populations, associated with ER difficulties and inflexibility [32–33] but not examined as the result of specific typologies of ER ability. Specifically, O’Bryan and colleagues [32] saw an association between specific ER difficulties (non-acceptance, limited ER strategies, and impulse control) and PTSD symptomology. It is therefore hypothesised that the latent typologies in ER flexibility may act as a contributory factor in both resilience ability and experience/severity of PTSD symptom distress.

As ‘resilience’ better describes a constellation of effects rather than a single, concrete mechanism, this study tested the above hypothesis using latent analysis, a technique which identifies unobserved underlying (latent) patterns in observed data. Latent profile/latent class analysis (LPA/LCA) uses individual endorsements/scores on a population level, identifying common groups (profiles or classes) within the population based on these. Turpyn and colleagues [34] have called using latent analysis in ER research a “person-centered approach”, focusing on the internal interactions of these variables within the individual and exploring similarities/differences in ER experience using profile/class description rather than relying on discreet scores [35–36]. Additionally, LPA/LCA has been used in this way to better explore PTSD symptom expression and experience in populations, demonstrating the variance of experience of PTSD distress [37–40].

This study utilised self-report data from a trauma-exposed sample to (1) identify potential ER flexibility typologies and (2) explore the relationship between potential latent ER flexibility typologies with both resilience and PTSD distress (as experienced through symptomology clusters of re-experiencing/intrusion, avoidance, negative cognition/mood, and hyperarousal). It was hypothesised that a number of typologies in ER flexibility will be observed through latent analysis and that typology membership would differentially relate to resilience and PTSD distress.

## Methods

### Study recruitment

A screener survey was launched on 14 October, 2021 on the Prolific survey platform (https://www.prolific.co) consisting of the Life Events Checklist for DSM-5 [41] (LEC-5). The inclusion criteria for the screen were for UK residents over the age of 18 who were fluent in English, and a quota was set for the screen to accept the first 1,003 participants before closing. This number was chosen based on costing resources and the assumption of attrition at each step of the survey procedure. Participants were paid £0.13 to complete the screen and those endorsing at least 1 trauma were invited to complete the full survey, meeting the trauma exposure criteria for the study. The full survey was hosted on the Qualtrics platform (https://www.qualtrics.com) and participants were compensated with £1.88 on completion. Participants could not progress to the next item/measure/page of the survey without completing all preceding items, resulting in no raw missing data. Ethical approval for this study was granted by the Queen’s University Belfast Engineering and Physical Sciences Faculty Research Ethics Committee (EPS 21_292) and all participants provided informed consent.

### Sample

Of the 1,003 participants who completed the screen, 885 endorsed at least 1 trauma, though 170 responses were excluded as the sole trauma endorsed was ‘any other very stressful event or experience’, leaving an eligible sample of *N* = 715 invited to participate in the full survey, of which *N* = 563 completed.

### Measures

The initial screen for trauma was done using the LEC-5 [41], a checklist of 17 categories of potentially traumatising events an individual may have experienced, including an item for ‘any other very stressful event or experience’. As used, the measure is binary (‘yes’ or ‘no’), with participants noting which events they have experienced, and here was scored as 1 = yes, 0 = no. The screen also included a follow-up item on which life event of those endorsed the participant considered the worst.

PTSD symptomology was measured with the PTSD Checklist for DSM-5 [25] (PCL-5), a measure consisting of 20 items to evaluate symptomology in alignment with DSM-5 criteria for PTSD. Respondents indicate the degree to which a symptom has caused them distress over the past month with answers on a 5-point Likert scale ranging from 0 (‘not at all’) to 4 (‘extremely’), with higher scores indicating more severe symptomology and a clinical threshold of probable PTSD caseness applied to scores of 34 and above [42]. The PCL-5 can also be broken down into symptom ‘clusters’: cluster B, intrusion/re-experiencing (items 1–5, cluster C, avoidance (items 6–7), cluster D, negative cognition/mood (items 8–14), and cluster E, hyperarousal (items 15–20). As used in this study, participants were asked to complete the PCL-5 as it related to their worst trauma endorsed in the screen and symptom cluster scores were utilised in the analysis. Internal consistency for the PCL-5 in this study was excellent (α = 0.95).

Emotional regulation ability was measured with the Difficulties in Emotion Regulation Scale—Short Form [43] (DERS-SF), an 18-item measure with 6 subscales describing different domains of ER difficulty: Strategies (limited access to ER strategies), Non-acceptance (non-acceptance of personal emotional responses), Impulse (difficulties with impulse control), Goals (difficulties in goal-oriented behaviour), Awareness (lack of emotional awareness), and Clarity (lack of emotional clarity). Participants are presented with a statement describing their emotions (example: “When I’m upset, I have difficulty concentrating.”) and asked to rate on a Likert scale ranging from 1 (‘almost never’) to 5 (‘almost always’) how often the statement matches their experiences. Higher overall and subscale scores are indicative of greater difficulties in emotional regulation. In this study, Items 1, 4, and 6 were reverse recoded before score calculation and the subscales were used as discreet variables. Internal consistency for the DERS was good (α = 0.88), with internal consistency for subscales ranging from acceptable to good (Awareness α = 0.77, Clarity α = 0.82, Goals α = 0.72, Impulse α = 0.71, Nonacceptance α = 0.72, Strategies α = 0.73).

Emotion regulation flexibility was measured using the Flexible Regulation of Emotional Expression Scale [44] (FREE). Participants are presented with 4 series of one-sentence scenarios and asked to rate on a Likert scale ranging from 0 (‘unable’) to 6 (‘very able’) how well they would be able to conceal or express positive or negative emotion in each hypothetical scenario. Higher overall scores indicate increased ability to express or supress external indicators of emotion, with 2 sub-scores describing expression and suppression ability. In this study, the subscales were used as discreet variables. Internal consistency for the FREE was good (α = 0.79) for both the overall measure and for the subscales (Expression α = 0.78, Suppression α = 0.72).

Context sensitivity was measured using the Context Sensitivity Index [45] (CSI) which uses short scenarios to measure sensitivity to the presence and absence of contextual cues in emotion-evoking situations. Participants are presented with each scenario (example: “A friend calls and asks you to do a favor for their partner, whom you don’t like.”), are asked to imagine themselves in that situation, and to rate their perception of the scenario in sub-items describing control, threat, external cooperation, and urgency using a Likert scale ranging from 1 (‘not at all’) to 7 (‘very much’). Sensitivity to the presence of contextual cues is measured by the Cue Presence Index (CPI) subscale (10 items) and sensitivity to their absence is measured by the Cue Absence Index (CAI) subscale (10 items), with higher scores representing higher sensitivity to the presence or absence of cues in emotion-evoking situations. For this study, the CPI and CAI were used as discreet variables.

Psychological resilience was measured with The Connor Davidson Resilience Scale [46] (CD-RISC-10), a 10-item measure adapted from the original 25-item measure [47] (CD-RISC). Participants are presented with a statement (example: “I am able to adapt when changes occur.”) and asked to rate on a Likert scale ranging from 0 (‘not true at all’) to 4 (‘true nearly all the time’) how truly the statement describes their experiences. Higher scores represent higher psychological resilience, and the total score was used in this study. Internal consistency for the CD-RISC in this study was good (α = 0.87).

Sociodemographic variables were measured with single items. Age was scored as a continuous variable. Participants were asked about their living arrangements (0 = living alone, 1 = living with others) and about their relationship status (recoded to 0 = single, 1 = currently in any type of relationship). Gender was used as a binary variable as a gendered effect would be tested in the analyses, with *N* = 5 gender variant/non conforming and *N* = 1 prefer not to say recoded as ‘not used’ (missing) and excluded listwise, and *N* = 1 transgender male recoded to male (0 = female, 1 = male).

### Analytical strategy

For use in these analyses, subscale scores (DERS-SF, FREE, and CSI) and cluster scores (PCL-5) were standardised using z-score values. All variables were normally distributed with no raw missing data and *N* = 6 excluded listwise for gender falling outside the traditional binary. A latent profile analysis (LPA) was conducted to determine if: (i) any latent groups were present in the population based on emotion regulation, emotion regulation flexibility, and context sensitivity, (ii) how many latent groups were present and, (iii) how these groups differed in the measured abilities.

Models for a 2 through 5-group solution were run, with 100 random starts used to avoid any solutions based on the local maxima. A range of fit indices were used to select the model of best fit. The Akaike information criterion [48] (AIC) functions as a quality determinant for model comparison and while it cannot provide the absolute quality of any given model, it can inform on the best among models, providing a log likelihood. The Bayesian information criterion [49] (BIC) relies on Bayesian inference but is susceptible to sample size, thus the sample-size adjusted BIC [50] (SSABIC) can be used in tandem to correct for larger populations. This analysis also utilised the Lo-Mendel-Rubin likelihood ratio test [51] (LMR-LRT), which compares a model with *k* number of profiles with a model featuring *k*—1 profiles, with a non-significant result indicating the model with *k*—1 profiles to be the better fit. Finally, the entropy criterion [52] was included, with a value ranging from 0 to 1 and a higher value indicating better fit.

Logistic regression was then performed to identify the extent to which sociodemographic variables (age, gender, relationship status, living alone), resilience scores, and PTSD symptom cluster scores predicted profile membership when compared to a reference class. All analyses were performed with IBM SPSS Statistics v26 [53] and MPLUS v8.1 [54].

## Results

Table 1 describes the sociodemographic characteristics of the sample, being predominantly white, female, with a mean age of 33.23 years (*SD* = 10.51, range = 18–75), living in England, in a relationship, employed, living in a home with others, with at least 1 qualification.

**Table 1:** Sociodemographic sample characteristics

	N	Population %
**Gender**		
Male	103	18.3%
Female	453	80.5%
Transgender male	1	0.2%
Gender variant/non-conforming	5	0.9%
Prefer not to say	1	0.2%
**Ethnicity**		
White	518	92.0%
Asian/multiple ethnic	11	2.0%
Asian/Asian British	20	3.6%
Black/African/Caribbean/Black British	10	1.8%
Other ethnic group	4	0.7%
**Location**		
England	474	84.2%
Scotland	50	8.9%
Wales	31	5.5%
Northern Ireland	8	1.4%
**Relationship status**		
Single/never married	196	34.8%
Married/living with partner	325	57.7%
Separated/divorced	34	6.0%
Widowed	1	0.2%
Prefer not to say	1	0.2%
Other	6	1.1%
**Employment**		
Unemployed	45	8.0%
Employed	416	73.9%
Student in higher education	63	11.2%
Retired	12	2.1%
Other	27	4.8%
**Living status**		
Does not live alone	503	89.3%
Living alone	60	10.7%
**Education**		
No qualifications	2	0.4%
GCSE’s	74	13.1%
A-Levels	103	18.3%
NVQ’s, CertHE’s, HNC, HND	111	19.7%
Bachelor’s degree	190	33.7%
Master’s degree	71	12.6%
Doctoral degree/PhD	12	2.1%

Descriptive statistics for the emotion regulation measures are presented in Table 2 below. Mean scores for the DERS subscales range from 5.75 (Impulse) to 9.63 (Goals), reflecting a population experiencing greater difficulties with Goals, Non-Acceptance, Awareness, and Strategies than Impulse and Clarity. This population showed greater ability in expressing emotion (34.61) than suppressing it (29.63), and slightly greater ability in detecting the presence of cues in situations (53.43) than in detecting their absence (51.65).

**Table 2:** Descriptives for emotion regulation measures

	N	Range	Mean (SD)
DERS Total	557	20–81	45.98 (12.98)
DERS Awareness	557	3–15	7.97 (2.74)
DERS Strategies	557	3–15	7.57 (3.06)
DERS Non-Acceptance	557	3–15	8.08 (3.33)
DERS Impulse	557	3–15	5.75 (2.90)
DERS Goals	557	3–15	9.63 (3.27)
DERS Clarity	557	3–15	6.95 (2.73)
FREE Total	557	26–96	58.73 (12.94)
FREE Expression	557	17–48	34.61 (6.04)
FREE Suppression	557	13–48	29.63 (6.47)
CSI Cue Presence	557	11–69	53.43 (7.24)
CSI Cue Absence	557	21–70	51.65 (7.70)

A latent profile analysis was performed to identify latent groups within the sample. Models were run sequentially from a 2-profile to 5-profile solution. In comparing the fit indices (Table 3), Entropy was highest for the 2-profile model and the LM-LRT was non-significant for the 3-profile model, indicating that the model *k*-1 is of better fit [44]. While all indices did decrease from a 2 to 3-profile model, the BIC only decreased by a small amount and is accepted as the best indicator of model fit [55]. Thus, the 2-profile model was selected as the model of best fit.

**Table 3:** Fit indices for latent profile analysis of emotion regulatory flexibility

	AIC	BIC	SSBIC	LRT (p)	VLMR-LRT	Entropy
1	24781.09	24911.09	24815.85	-	-	-
**2**	**13954.46**	**14125.75**	**13998.75**	1254.98 (0.00)***	-7212.42 (0.00)***	**0.87**
3	13644.33	13901.26	13710.81	347.36 (0.18)	-6587.09 (0.01)*	0.84
4	13530.55	13873.14	13619.19	152.56 (0.20)	-6402.21 (0.04)*	0.84
5	13460.26	13888.49	13571.06	109.42 (0.19)	-6326.42 (0.08)	0.85

AIC = Akaike information criterion, BIC = Bayesian information criterion, SSBIC = sample size adjusted Bayesian information criterion, LRT = Lo-Mendell-Rubin adjusted likelihood ratio test; VLMR-LRT = Vuong-Lo-Mendell-Rubin adjusted likelihood ratio test; model of best fit in bold, - = not provided on 1-class solution

**Fig. 1:**
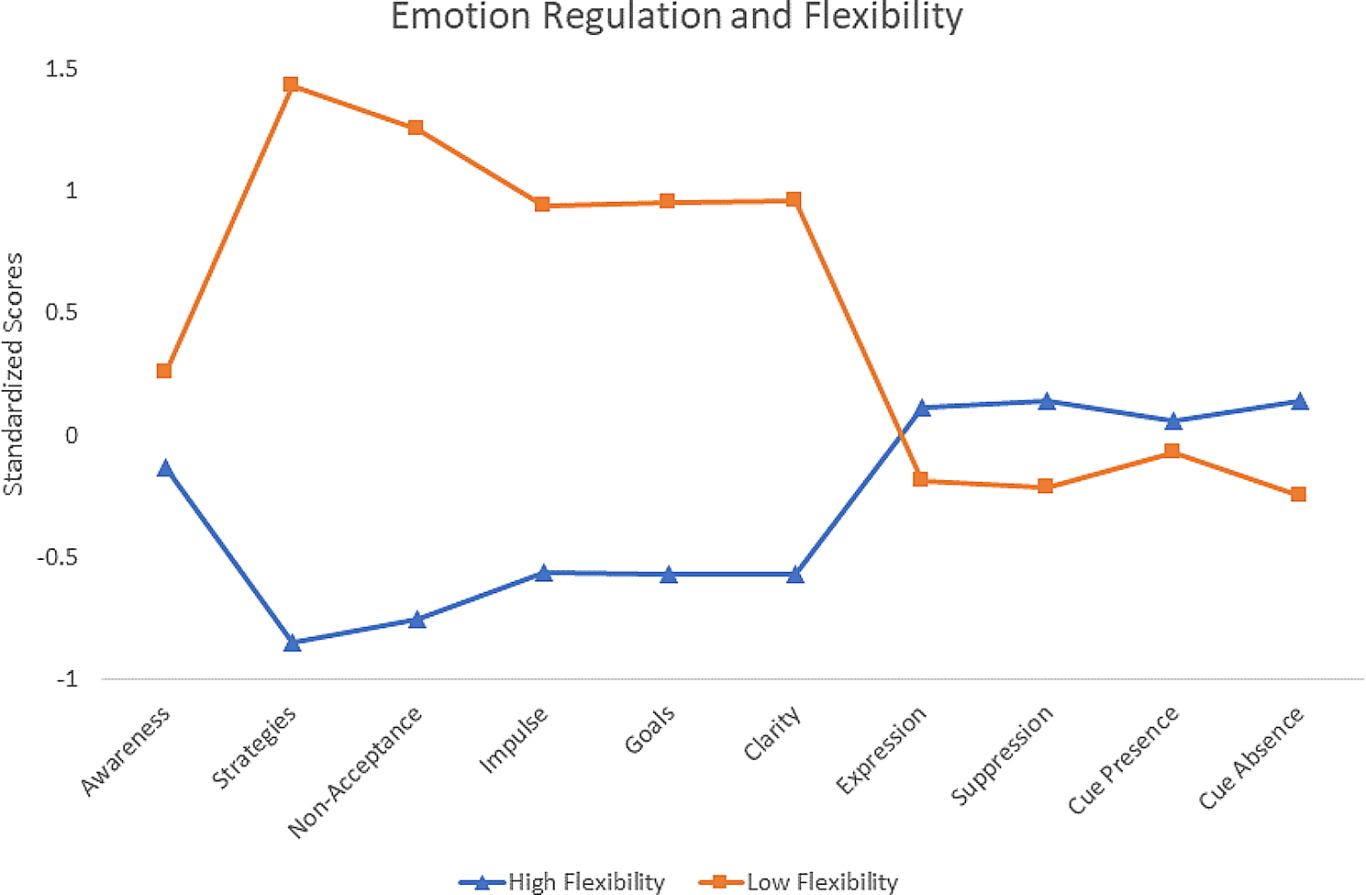
Endorsement plot of high and low flexibility classes

The 2 profiles are described in Fig. 1 below, with the Low Flexibility group (*N* = 206, 37.7%) showing increased difficulties across all DERS subscales, particularly Strategies and Non-Acceptance, and decreased flexibility skills. The High Flexibility group (*N* = 341, 62.3%) shows decreased difficulties in emotional regulation, particularly Strategies and Non-Acceptance, and increased flexibility skills.

Table 4 shows the group endorsements for all emotion regulation variables and the difference (in standard deviation) between them. Differences are quite profound for Strategies (2.26) and Non-Acceptance (2.00), and to a lesser extent, Clarity (1.52), Goals (1.51), and Impulse (1.50).

**Table 4:** Class comparison of high and low flexibility classes

	High flexibility class	Low flexibility class	Difference (SD)
DERS Awareness	-0.13	0.25	0.38
DERS Strategies	-0.84	1.42	2.26
DERS Non-Acceptance	-0.75	1.25	2.00
DERS Impulse	-0.56	0.94	1.50
DERS Goals	-0.56	0.95	1.51
DERS Clarity	-0.56	0.96	1.52
FREE Expression	0.11	-0.18	0.29
FREE Suppression	0.14	-0.21	0.35
CSI Cue presence	0.05	-0.07	0.12
CSI Cue absence	0.14	-0.24	0.38

A logistic regression was performed to determine what factors were associated with Low Flexibility group membership when compared to the High Flexibility group as a reference class (Table 5). Membership in the Low Flexibility group was associated with younger age, male gender, lower resilience, and with the PTSD symptom clusters of negative cognition/mood and hyperarousal.

**Table 5:** Logistic regression odds ratios for association variables

	Estimate	S. E.	Odds Ratio (p)	95% CI
Age	-0.05	0.02	-0.05 (0.001)**	-0.01–0.11
Gender	0.89	0.59	0.89 (0.03)*	-1.08–1.40
Relationship	0.08	0.35	0.08 (0.81)	-0.98–0.51
Living alone	-0.26	0.55	-0.26 (0.61)	-1.21–1.12
Resilience	-0.93	0.02	-0.93 (0.00)***	0.05–1.17
Re-experiencing/intrusion	-0.41	0.23	-0.41 (0.85)	-0.61–0.35
Avoidance	-0.20	0.24	-0.20 (0.40)	-0.27–0.71
Negative cognition/mood	1.40	0.29	1.40 (0.00)***	-2.12–1.85
Hyperarousal	0.96	0.27	0.96 (0.00)***	-1.71–1.01

*p < 0.05; **p < 0.01; ***p < 0.001

## Discussion

The current study had two aims; (1) to identify potential ER flexibility typologies and (2) to explore the relationship between potential latent ER flexibility typologies in that sample with both resilience and PTSD distress (as experienced through symptomology clusters of re-experiencing/intrusion, avoidance, negative cognition/mood, and hyperarousal). Concerning the first aim, an LPA was run which concluded the optimal fitting model comprised two latent profiles of ER flexibility. Two groups were identified, a Low Flexibility and a High Flexibility group (Fig. 1). While the two groups had similar endorsements in expression/suppression of emotion and sensitivity to cue absence/presence, they diverged in endorsement of emotion regulation. Specifically, the differences between the two groups were the greatest in access to Strategies and levels of Non-Acceptance and to a lesser extent, Impulse, Goals, and Clarity (Table 4). These profiles seemed to ‘mirror’ each other across the mean, representing with the Low Flexibility group’s deficits reflected by the High Flexibility group’s abilities. Concerning the second aim, when compared against members of the High Flexibility group, members of the Low Flexibility group were more likely to be younger, male, and show lower resilience scores. This group membership was also associated with increased experiences of negative cognition/mood and hyperarousal.

The largest variance between the two groups was in ER difficulties, which apart from lack of emotional Awareness, showed differences as profound as > 2 SD (Table 4). This was particularly true for a lack of ER Strategies and Non-Acceptance of emotional responses, replicating O’Bryan and colleagues’ [32] findings, reporting the association of Non-Acceptance with hyperarousal and negative cognition/mood. This paints a picture of a typology defined more by difficulties in ER regulation than by contextual sensitivity and flexible expression. Following the sequential ER flexibility model, the DERS subscales describe a failure of the repertoire and feedback monitoring steps, with the lack of coping strategies compounding the disconnect between the individual and their emotional responses and hampering impulse-control and goal-oriented behaviour. While the Low Flexibility group’s contextual awareness and flexibility in expressing/repressing emotions was also lower than the High Flexibility group, the findings here show that ER difficulties were the defining features in this population.

Low Flexibility group membership was associated with lower trait resilience and with the PTSD symptom clusters of negative cognition/mood and hyperarousal. These symptom clusters account for (respectively): amnesia, negative beliefs, blame, negative feelings, loss of interest, detachment or estrangement, and numbing, and irritability/aggressive behaviour, reckless behaviour, hypervigilance, startle response, concentration, and sleep impact [56]. It is important to note the significance of increased negative cognition/mood and hyperarousal symptomology but not re-experiencing/intrusion or avoidance, and this maps onto the ER flexibility sequence framework. Re-experiencing/intrusion can be described as cognitive processes potentially related to memory fragmentation around the event and avoidance as an internal protective measure against potential re-traumatisation, while negative cognition/mood and hyperarousal could represent an increase in distress based in inappropriate/maladaptive coping strategies due to decreased coping repertoire and/or the inability to re-evaluate and re-select appropriate strategies.

In this sample, an insufficient repertoire of effective coping strategies and individual lack of emotional awareness predicted PTSD symptomology which can be interpreted as a ‘vicious circle’ of negative emotions, inability to adequately cope, and maladaptive behaviour potentially leading to an increase in negative emotions. As this study was cross-sectional, future studies should focus on a longitudinal model and examining changes in symptomology over time, as ER flexibility changes may impact PTSD distress, especially if the individual sought treatment.

Differences in the DERS subscales between the two groups are larger than those found in the CSI subscales (Table 4) and while Cue Presence is nearly equivalent (0.12 SD) for the Low and High Flexibility groups, the divergence for Cue Absence was higher (0.38 SD). Context sensitivity describes the ability of the individual to take in contextual cues from the environment during the event, developing an understanding of the situation which guides their reaction in the moment but also all consecutive reactions in the event aftermath, including selecting an appropriate coping strategy [45]. While recognising cues is important, recognising the lack of cues is equally so, especially when it influences dependant actions. Threat is a good example, as responding to the presence of threat in the situation will inform actions, reactions, and coping in a manner which is (hopefully) adaptive/appropriate to the situation, but failing to notice a lack of threat could lead the individual to actions, reactions, and coping which are maladaptive/inappropriate to the situation. Thus misinterpretations of contextual cues could misinform all ‘downstream’ functions of ER flexibility, impacting on post-event distress.

Finally, and perhaps most importantly, the Low Flexibility group was the smaller of the two (*N* = 206, 37.7%) and group members were more likely to be male. The sample demographic was predominantly female (*N* = 453, 80.5%) and while there are established gender differences in PTSD symptomology and distress [30–31], few studies explore the peritraumatic ER differences of the participants and the cultural contexts of gender socialisation in ER ability [57]. Difficulties in ER are not gender-exclusive though previous studies have reported gender-based trends regarding ER ability [58–59]. For example, Goubet and Chrysikou [60] described women as having access to a more diverse repertoire of strategies and being more flexible in implementation than the men in their study, suggesting a crucial difference in gender socialisation surrounding emotional awareness, expression, and flexibility. Future research should take gendered socialisation into account.

### Strengths and limitations

These findings must be taken alongside the study’s limitations. Self-report data was utilised, which always carries the risk of social desirability bias [61]. The study population was not a representative sample and therefore the results cannot be generalised to the superordinate UK population. As recruitment was done through the Prolific survey platform and participation limited to platform members, it is possible that results are associated with specific qualities of individuals (or groups of individuals) more likely to participate in online research or to participate on certain platforms [62]. Additionally, the use of membership-only online recruitment may not include those too unwell to participate. While an IP-lock was used to ensure that respondents were participating from the UK, more sophisticated anti-bot measures were not used, nor were attentional checks. ER ability and flexibility was determined here by the use of multiple measures in aggregate as there is no one absolute test/scale/tool for measuring these constructs. While the LEC-5 does have an item querying ‘any other stressful event or experience’, those who only endorsed this item in the screener survey were not invited to complete the full survey, as DSM-5 criteria for PTSD does not consider these experiences as ‘valid’ for assessing PTSD [63–66]. PTSD symptom cluster scores only described distress and did not represent a diagnosis of PTSD, nor was PTSD caseness described. The amount of time which had passed since the trauma exposure was not asked, meaning that no conclusions can be drawn regarding any additional effect of ER ability/flexibility over time. Cumulative trauma, trauma type, participants’ social support/support network, and trauma type were not considered, and may bring nuance to these findings in future replications. Finally, the data used here was cross-sectional, which does not and cannot imply temporal causality, only an association, and does not allow for longitudinal tracking of this dynamic process.

This study does have several notable strengths, however. First, the screener design of the study ensured that only participants who were trauma-exposed were invited to the full survey, second, the use of latent analysis allowed for the exploration of unobserved typologies in the population which have been relatively underexplored in studies using the ER flexibility sequential model, and finally, the parsimonious design encourages replication studies across larger and more diverse populations.

### Impact and implications

These findings have important clinical implications. ER flexibility represents the aggregate of several skills, some of which can be improved through both clinical [67–68] and non-clinical practice [69]. This raises the possibility of bespoke pre-trauma strategies to bolster ER flexibility in individuals. Such strategies could be educational in nature, building ER flexibility in children and adolescents, for example, or informal initiatives offered for adults. Formal therapeutic services utilising ER ability/flexibility with a goal of impacting the severity of PTSD symptomology and distress following trauma exposure do exist, and would benefit from research exploring the functionality of specific underlying psychological mechanisms. Additionally, the association between ER inflexibility and specific symptom clusters could imply the development of therapy initiatives to ‘target’ this distress by bolstering the associated ER skills. Future research should focus on the relationship between ER flexibility and PTSD in larger and more diverse populations, as well as interventions to test if building/improving ER flexibility post-exposure improves symptomology or distress.

## Conclusions

It is evident that ER flexibility and resilience contribute to the experience of PTSD symptomology and distress following exposure to a traumatic event. While it is reductive to think of resilience as an absolute typology, it is more likely that latent typologies in ER flexibility have a significant impact on both resilience and PTSD when viewed through the ER flexibility sequential model.

### Electronic supplementary material

Below is the link to the electronic supplementary material.

Supplementary Material 1

## Data Availability

The datasets generated and/or analysed during the current study are not publicly available due embargo but are available from the corresponding author on reasonable request.

## Declarations

### Compliance

The authors state that all methods were carried out in accordance with relevant guidelines and regulations.
