# Supplementary material for: Flexible emotional regulation typology: associations with PTSD symptomology and trait resilience
Source: BMC Psychol. 2024 Feb 16;12:79. doi: 10.1186/s40359-024-01573-4 (PMC10874029; doi:10.1186/s40359-024-01573-4)
Supplement: Supplementary file 1 — Supplementary Material 1 [file 40359_2024_1573_MOESM1_ESM.docx]

**Supplementary Documentation**

***Table SD1. Correlation matrix of emotion regulation variables and PTSD symptom clusters***

|  | **Intrusion** | **Avoidance** | **Neg. cognition & mood** | **Hyperarousal** |
| --- | --- | --- | --- | --- |
| DERS Awareness | 0.09 | 0.12 | 0.19 | 0.12 |
| DERS Strategies | 0.59 | 0.41 | 0.68 | 0.66 |
| DERS Non-Acceptance | 0.49 | 0.39 | 0.61 | 0.54 |
| DERS Impulse | 0.40 | 0.27 | 0.43 | 0.53 |
| DERS Goals | 0.41 | 0.33 | 0.49 | 0.51 |
| DERS Clarity | 0.40 | 0.28 | 0.51 | 0.48 |
| FREE Expression | 0.01 | 0.01 | -0.02 | -0.03 |
| FREE Suppression | -0.03 | -0.03 | -0.06 | -0.09 |
| CSI Cue presence | 0.02 | 0.05 | 0.01 | -0.02 |
| CSI Cue absence | -0.18 | -0.14 | -0.15 | -0.11 |
